# Atypical Autoimmune Encephalitis With Neuropil Antibodies Against a Yet Unknown Epitope

**DOI:** 10.3389/fneur.2019.00175

**Published:** 2019-03-05

**Authors:** Sharmili Edwin Thanarajah, Harald Prüss, Clemens Warnke, Michael T. Barbe, Michael Schroeter, Gereon R. Fink, Oezguer A. Onur

**Affiliations:** ^1^Department of Neurology, Faculty of Medicine and University Hospital of Cologne, University of Cologne, Cologne, Germany; ^2^Max Planck Institute for Metabolism Research, Cologne, Germany; ^3^Department of Neurology and Experimental Neurology, Charité - Universitätsmedizin Berlin, and German Center for Neurodegenerative Diseases (DZNE) Berlin, Berlin, Germany; ^4^Cognitive Neuroscience, Institute of Neuroscience and Medicine (INM-3), Research Center Jülich, Jülich, Germany

**Keywords:** encephalitis, cerebellitis, autoimmune, neuropil, plasmapheresis

## Abstract

Autoimmune encephalitis often causes acute psychiatric symptoms and epileptic seizures. However, it is becoming increasingly clear that depending on the target antigen both symptoms and disease severity may vary. Furthermore, the identification and characterization of antibody subtypes are highly relevant for personalizing the treatment and to prevent relapses. Here we present an atypical case of encephalitis with cerebellar and temporal dysfunction but without seizures associated with high levels of cerebrospinal fluid neuropil antibodies against a yet unknown epitope on the neuronal surface in the cerebellum, hippocampus, thalamus, and the olfactory bulb. We treated the patient successfully with corticosteroids, plasmapheresis, and rituximab.

## Introduction

Autoimmune-mediated encephalitis is commonly associated with acutely emerging psychiatric symptoms and seizures refractory to antiepileptic treatment (1, 2). Evidence increases that, depending on the underlying immune processes and the target antigen, structures of the central nervous system beyond the limbic cortex can be affected, leading to a great spectrum of disease presentation and severity (3, 4).

New insights into the mechanisms underlying antibody associated encephalitis highlight the importance of early diagnosis and antibody characterization using both specialized serum/cerebrospinal fluid (CSF) panels and immunohistochemistry to initiate an effective, personalized treatment regime that addresses both the prevention of disease progression and relapses (5, 6). However, due to the varying clinical, imaging, and serum/CSF manifestations, the early diagnosis and the appropriate treatment still remain a great challenge.

Here we present an atypical case of encephalitis with cerebellar and temporal dysfunction but without seizures associated with high levels of CSF neuropil antibodies against a yet unknown epitope on the neuronal surface in the cerebellum, hippocampus, thalamus, and the olfactory bulb. We treated the patient successfully with corticosteroids, plasmapheresis, and rituximab.

## Case Study

A 67-years old lady was admitted to our intensive care unit (ICU) after requiring intubation for respiratory failure secondary to aspiration pneumonia and decreased consciousness.

Past medical history revealed that 6 months earlier she had been admitted to her local hospital with features of delirium (disorientation, agitation, poor memory functioning) and a cerebellar syndrome with intention tremor, severe postural instability, and dysarthria.

**Figure 1:**
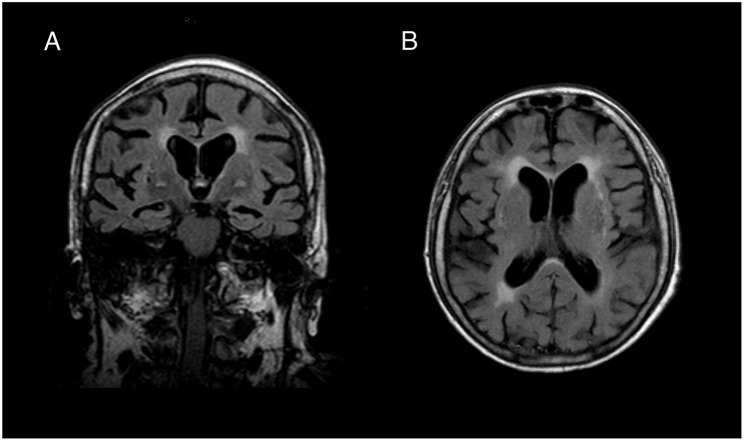
Brain MRI. **(A)** Coronal and **(B)** transversal slice of T1-weighted brain imaging revealed moderate temporal lobe atrophy (medial temporal lobe atrophy score 2) and white matter lesions (Fazekas grade 2). We did not find any indication for inflammatory processes in FLAIR, T2- and Diffusion weighted sequences.

In the local hospital, brain MRI showed a moderate temporo-parietal atrophy (medial temporal lobe atrophy score 2, Figure 1). Repeated EEGs did not reveal epileptic activity. Routine blood and CSF analysis (cell count, glucose and protein level) were normal and investigation for infectious pathogens (varicella zoster virus, herpes zoster virus, cytomegalovirus, mumps, rubella, borrelia burgdorferi, treponema pallidum) were negative. Screening for occult malignancy (CT of thorax and abdomen) showed a right ovarian enlargement, but the surgical biopsy did not reveal a neoplastic process. Suspecting an autoimmune pathogenesis, a 5-day course of intravenous high-dose prednisolone (1,250 mg per day) and a 5-day course of intravenous immunoglobulin (IVIg) (20 g IVIg per day) were applied, leading to complete remission of dysarthria and truncal ataxia and partial remission of the delirium. The patient was subsequently transferred to the rehabilitation facility, where the cerebellar symptoms and the level of consciousness deteriorated significantly. She required oropharyngeal intubation due to respiratory failure and was admitted to our ICU.

Upon admission, the patient showed a septic shock due to aspiration pneumonia requiring vasopressor drugs. Following antibiotic treatment (piperacilin/tacobactam and clarithromycin) the vital signs returned to the normal ranges. After sedation was stopped the patient was in a minimally conscious state. Pupillary and corneal reflexes were regular. Gag and cough reflexes were absent.

Brain MRI did not provide new findings. EEG on admission, and repeated EEGs during the stay did not display epileptic activity. The CSF work-up showed normal cell count, glucose and protein levels. Tau- and Phospho-Tau-protein level were slightly elevated while ß-Amyloid and Amyloid-ratio were decreased (Tau 622 pg/ml, Phospho-Tau 71 pg/ml, Amyloid ratio 0.05 pg/ml, Amyloid ß1-42 393 pg/ml). Identical oligoclonal bands were found in serum and CSF. Screening for common anti-neuronal and anti-neuropil antibodies was negative (VGKC, GAD65, NMDAR, GABABR, IgLON5, AMPAR2, DPPX, LGI1, CASPR2, Glycin-receptor, mGluR5, Amphiphysin, CV2/CRMP5, Ma2/Ta, Ri, Yo, Hu, Recoverin, Sox1, Titin, Zic4, DNER/Tr).

**Figure 2:**
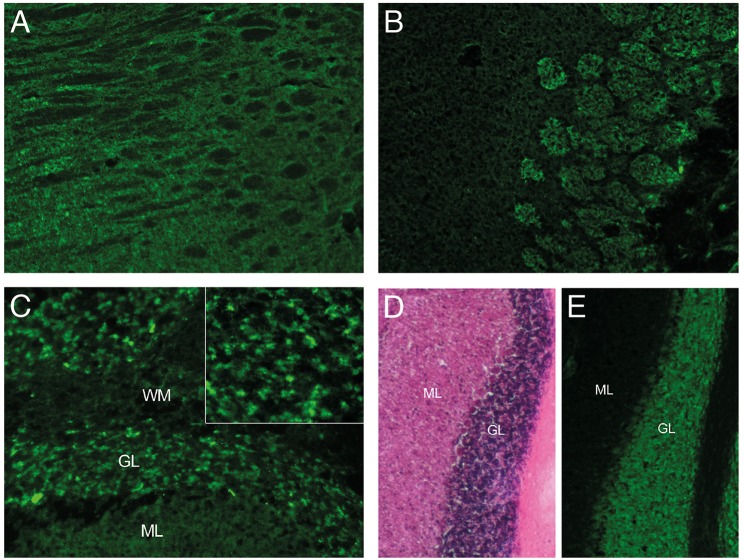
Immunoreactivity of CSF. Indirect immunostaining of CSF with unfixed mouse brain sections showing signal to neuropil in the **(A)** thalamus, **(B)** olfactory bulb, and **(C)** cerebellum. CSF reactivity is restricted to a subset of granule cells (GL) in the cerebellum (C, insert), as compared to the dense neuronal layer seen with H&E staining **(D)** or the neuronal marker NeuN **(E)** of adjacent sections. (ML, molecular layer; WM, white matter).

Increased Tau-protein level, decreased level of Amyloid-ratio and temporo-parietal atrophy suggested a pre-existing Alzheimer's disease. Still suspecting an autoimmune process, CSF and serum were incubated on mouse brain sections revealing strong antibody reactivity on neuropil and neuronal surface targeting a still unknown epitope in the cerebellum, thalamus, hippocampus, and the olfactory bulb (Figure 2). In the cerebellum, antibody reactivity was predominant on granular cells. Interestingly, the patient's CSF stained only a fraction of granule cells, which further underlines specificity as it is similarly known from other autoimmune encephalitides, such as NMDAR or GABAaR encephalitis.

The patient received intravenous high-dose prednisolone for 5 days and 10 courses of plasmapheresis. The clinical status improved significantly: She was able to follow commands and communicate and did not show psychotic symptoms. For long-term disease modifying treatment, we then introduced intravenous rituximab (500 mg). We recommended a clinical reevaluation and repeated screening for occult malignancy with CT of thorax and abdomen in a year.

## Discussion

Our case illustrates that autoimmune-mediated encephalitis may present with a broad spectrum of symptoms depending on the target antigen. Our patient developed a fast progressing condition with severe memory deficits, behavioral changes, ataxia and deterioration of consciousness, but no epileptic seizures. Correspondingly, we detected highly reactive antibodies to a yet unknown epitope on neuropil and neuronal surface in the hippocampus, cerebellum, and thalamus suggesting autoimmune mediated encephalitis although CSF workups did not detect lymphocytic pleocytosis.

In contrast to antibodies targeting intraneuronal antigens, that are not pathogenic and suggest a T-cell mediated process, antibodies against neuropil and neuronal surface proteins are often pathogenic (7), but cause reversible changes and limited neuronal death (8). The surface binding pattern of the antibodies in our case suggests pathogenicity involving auto-reactive B-cells and antibody-secreting plasma cells, even though the undetermined epitopic target precludes final proof. Remarkably, this type of encephalitis has been demonstrated to be more responsive to immunotherapy (5, 7, 9). Hence, a detailed classification of antibody-associated encephalitides may guide the individual therapeutic management and thereby help to improve the outcome. However, unknown and rare antibodies will be missed by most currently available commercial tests, particularly if only the serum is analyzed. Hence, a comprehensive CSF and serum workup using immunohistochemistry seems warranted.

So far, no evidence-based guidelines exist for the optimal therapeutic approach to autoimmune encephalitis associated with anti-neuropil antibodies. We applied corticosteroids, IVIg and plasmapheresis and found the strongest clinical improvement after combined treatment with corticosteroids and plasmapheresis. This observation is in line with a recent review on NMDAR-encephalitis (10), the most common anti-neuropil antibody associated encephalitis. In case of a relapse or delayed diagnosis, the escalation to second-line treatment with rituximab, cyclophosphamide, or both is recommended (11). We applied intravenous rituximab to selectively deplete circulating CD20-B-cells (12), and achieve long-term prevention to relapses. Due to its B-cell specificity rituximab has a better safety profile as compared to anti-metabolites, such as cyclophosphamide, which inhibit cell-proliferation of both B- and T-cells and have more toxic side effects such as infertility, myelosuppression, and an increased malignancy risk (5, 13).

Our case demonstrates that autoimmune-mediated encephalitis needs to be considered in any condition with fast progressing central nervous symptoms even if the CSF analysis does not reveal elevated cell counts or classical antibodies and brain imaging does not detect changes indicating inflammatory processes.

## Ethics Statement

The patient gave written consent in accordance with the Declaration of Helsinki.

## Author Contributions

SET, CW, MS, MB, GF, and OO provided clinical care. SET and OO planned the case report and interpreted the data. HP analyzed the CSF data. SET drafted the manuscript. SET, CW, MS, MB, GF, and OO revised the manuscript. All authors approved the final version of the manuscript.

### Conflict of Interest Statement

The authors declare that the research was conducted in the absence of any commercial or financial relationships that could be construed as a potential conflict of interest.
